# Oral Manifestations of Parry-Romberg Syndrome: A Case Report

**DOI:** 10.7759/cureus.63984

**Published:** 2024-07-06

**Authors:** El mehdi Hariri, Mohamed Sellouti, Hind Ramdi

**Affiliations:** 1 Pediatric Dentistry, Faculty of Dental Medicine of Rabat, Mohammed V University in Rabat, Rabat, MAR; 2 Pediatric Dentistry, Mohammed V Military Hospital of Rabat, Rabat, MAR; 3 Neonatology, Faculty of Medicine and Pharmacy of Casablanca, Hassan II University of Casablanca, Casablanca, MAR

**Keywords:** dark scar, dental abnormalities, oral manifestations, hemifacial atrophy, parry-romberg syndrome

## Abstract

Parry-Romberg syndrome is a rare acquired disorder characterized by unilateral idiopathic progressive atrophy of the skin and soft tissues of the face, resulting in a sunken appearance. The muscles, cartilage, and underlying bony structures may also be affected. The etiology remains unclear and is based on several hypotheses. The incoherence of atrophy and the development of associated symptoms make the diagnosis, prognosis, and management of patients difficult. Here, we report the case of a 10-year-old boy who presented to the Department of Pediatric Dentistry at the Mohamed V Military Training Hospital in Rabat with progressive left hemifacial atrophy and was diagnosed by a pediatric rheumatologist as having Parry-Romberg syndrome. On extraoral examination, the patient presented a slight facial asymmetry and a small, dark, linear scar in the left zygomatic region. Intraoral examination revealed a left lateral open bite and atrophy of the left side of the tongue. Panoramic radiography showed incomplete eruption of the left mandibular first and second premolars (34/35), with significant root atrophy giving a narrowed appearance to the corresponding pulp chambers confirmed on retro alveolar radiographs. The mandible had a slightly reduced ramus height on the affected side confirming the patient's facial asymmetry. A better understanding of this syndrome will help to improve oral care in young patients.

## Introduction

Parry-Romberg Syndrome, also known as progressive hemifacial atrophy, is a rare degenerative disorder characterized by unilateral atrophy of facial tissues including muscles, bones, skin, and cartilage [1,2]. This can lead to aesthetic concerns, as well as functional and psychological challenges due to facial asymmetry. It was first documented by Parry in 1825, and subsequently by Romberg in 1846 with unclear etiology, despite extensive study [3,4]. Historically, it has been debated whether progressive hemifacial atrophy is a form of linear scleroderma, known as morphea en coup de sabre [5]. Other authors are clear that it is an intrinsically different process or on a spectrum. It is now accepted that these two conditions are a part of the spectrum of localized scleroderma and often co-exist [6]. While dermatologic and ophthalmologic manifestations are well-documented, oral manifestations are less frequently reported [7,8].

Below, we report a case of oral manifestations observed in a young Moroccan patient diagnosed with Parry-Romberg Syndrome by a pediatric rheumatologist, which was confirmed by positive serological tests for antinuclear antibodies directed against double-stranded deoxyribonucleic acid and mitochondria, and by a biopsy revealing the presence of morphea.

## Case presentation

**Figure 1:**
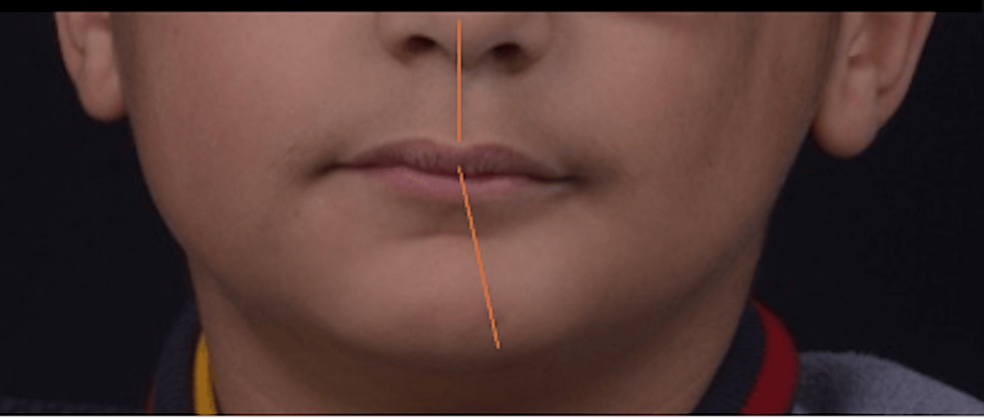
Facial asymmetry in relation to the median sagittal plane

We received a 10-year-old Moroccan boy in the Department of Pediatric Dentistry at Mohamed V Military Hospital in Rabat with complaints of progressive hemifacial atrophy and difficulty chewing on the left side. The patient's general health was good, and we found no pathological history to explain the facial asymmetry (Figure 1). No family history, particularly no siblings with the disease and our patient did not report the use of any medication prior to the discovery of the condition.

The diagnosis of Parry-Romberg syndrome was made by a paediatric rheumatologist after carrying out two tests: serological tests for antinuclear antibodies directed against double-stranded deoxyribonucleic acid and mitochondria and a biopsy which revealed the presence of morphea. Several other diagnoses were suggested prior to testing, including hemifacial hypoplasia, scleroderma, oculoauriculovertebral-related disorders, lipodystrophy, and fat necrosis.

**Figure 2:**
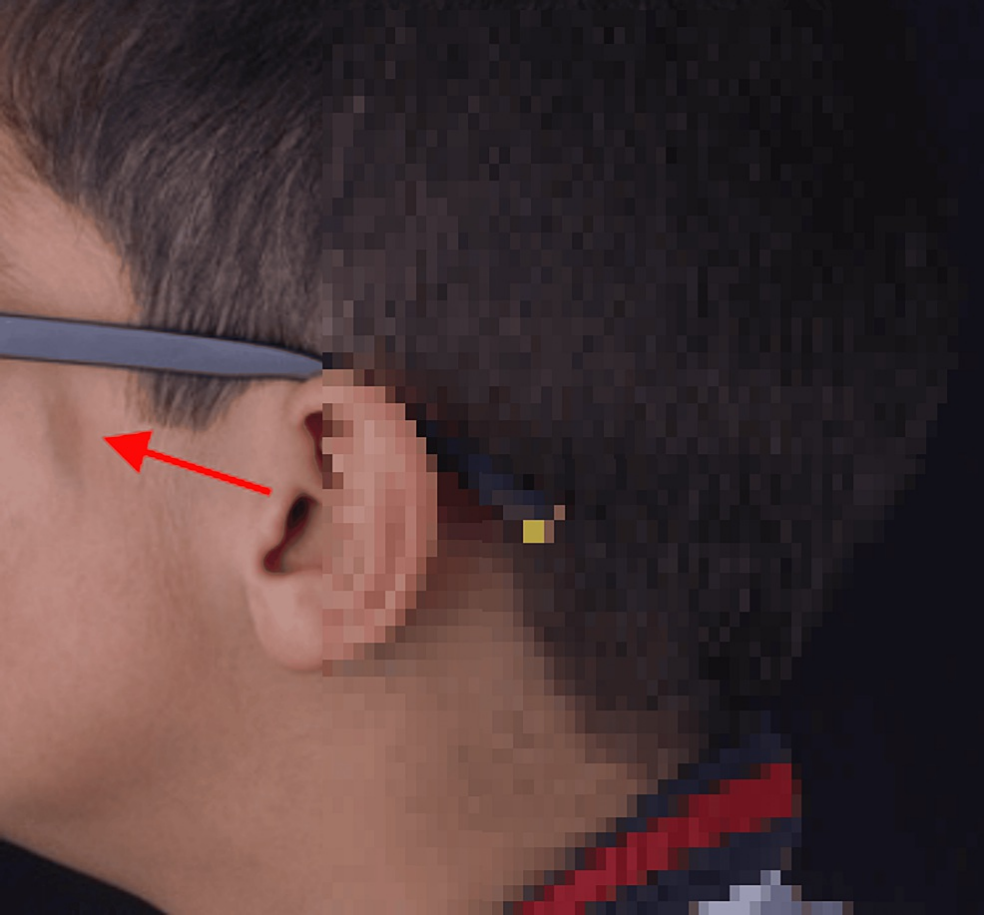
Morphea en coup de sabre in the left zygomatic region

On extraoral examination, slight asymmetry was observed on the left side of the face, giving the appearance of a mandibular deviation, along with a small dark linear scar in the left zygomatic region, called morphea en coup de sabre (Figure 2).

**Figure 3:**
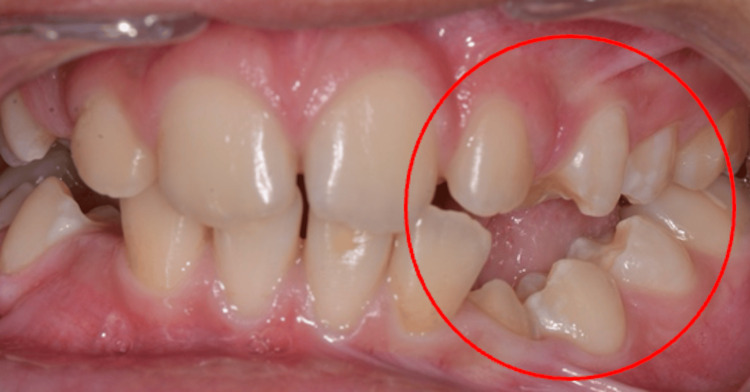
Left unilateral open bite on the affected side

**Figure 4:**
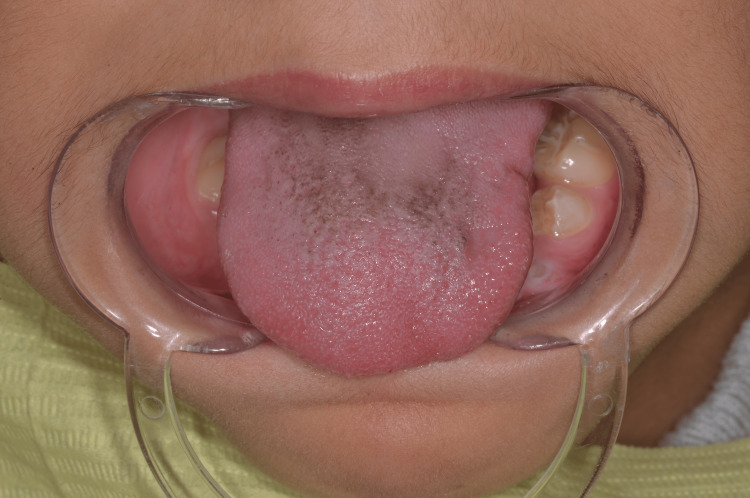
Atrophy of the left lateral border of the tongue

During the intraoral examination, several notable findings were observed. These include a left lateral open bite due probably to incomplete eruption of the first and second left mandibular premolars (34/35) (Figure 3). The patient also has atrophy of the left side of the tongue, which gives the appearance of an asymmetrical tongue in relation to the median sagittal plane, resulting in significant difficulty in moving the tongue from right to left (Figure 4). Additionally, there are signs of incipient temporomandibular joint dysfunction, particularly a slight left deviation of the mandible on opening.

**Figure 5:**
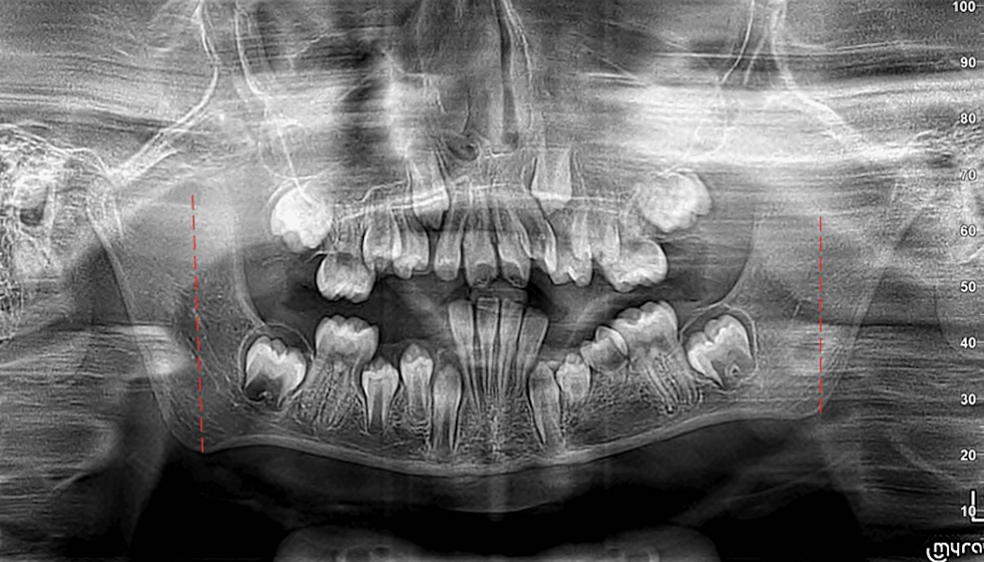
Decreased ramus height on the left side

**Figure 6:**
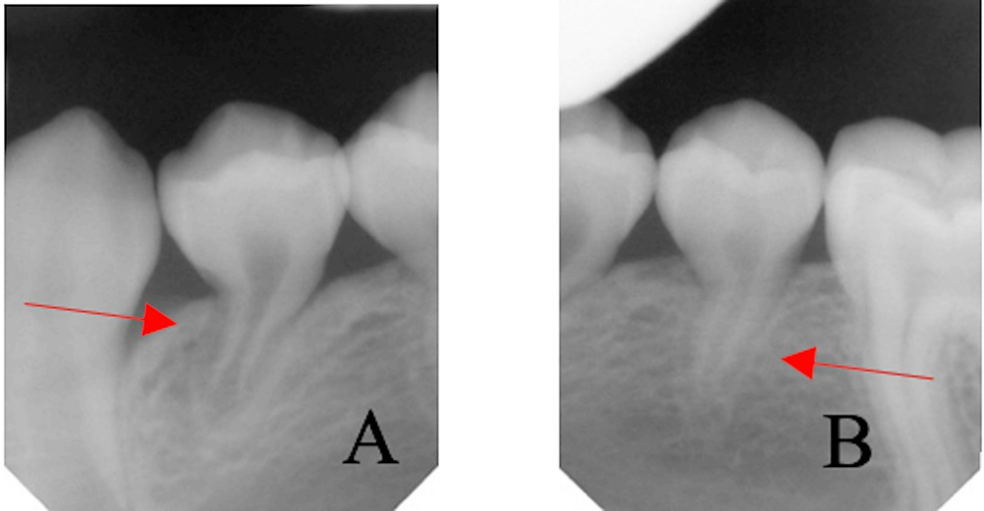
Root atrophy of the first and second left mandibular premolars Premolars 34 (A) and 35 (B) with the narrowed appearance of the corresponding pulp chambers.

The panoramic radiograph (Figure 5) shows the incomplete eruption of the mandibular first and second premolars on the affected side (34/35), with significant root atrophy giving a narrowed appearance to the corresponding pulp chambers (Figures 6A, 6B). This appearance is very visible in the retro alveolar radiographs. The mandible has a slightly reduced ramus height on the affected side, which also confirms the patient's facial asymmetry and slight left deviation of the mandible on opening (Figure 5).

At this stage, our patient is only being treated with corticosteroids (oral prednisolone at a 1 mg/kg/day dose) to suppress the activity of the immune system in order to slow the progression of the condition and improve his quality of life. Our patient's parents have also reported a significant increase in the child's weight following treatment with no clear improvement in hemifacial atrophy. In order to have a positive impact on the patient's psychology, the parents have explained to the child that this is a temporary problem that will improve with time.

## Discussion

This case illustrates some of the oral manifestations associated with Parry-Romberg syndrome, which can have a significant impact on the patient's oral health and quality of life. The condition is more common in females, with a predilection for the left side of the face [8]. The prevalence rate is estimated to be at least one in 7,000 in the general population. The condition is most common in the first and second decades of life [9]. The progression of this disorder can stabilize at any stage of growth and development. The skin on the affected side has a resembling scleroderma and may be pigmented, as in our case (Figure 2). In some patients, a linear scar may mark the border between normal and abnormal skin, called morphea en coup de sabre [10,11].

Several factors have been identified as potentially involved in the pathogenesis of Parry-Romberg syndrome, including disturbances in fat metabolism, trauma, viral infections, heredity, endocrine disturbances, and autoimmunity [12]. However, none of these theories provides a satisfactory explanation for the condition, and the pathogenesis of Parry-Romberg syndrome remains unresolved. Recently, there have been reports in the literature of extracutaneous involvement, which suggests that this is not only a cutaneous disease [13].

Although the intraoral soft tissues and masticatory muscles are affected, normal functions such as speech and deglutition are not affected [14,15]. There may be deformity of the mouth and nose on the affected side, as well as marked atrophy of the tongue papillae and the left lateral border, as observed in our case (Figure 4). Lingual atrophy and oral mucosal changes may predispose the patient to periodontal disease and oral mucosal damage, emphasizing the importance of regular maintenance and monitoring of oral hygiene. Root atrophy, defects in tooth eruption, and delayed tooth formation and maturation can also be seen in Parry-Romberg syndrome, as in our case (Figures 6A, 6B). However, the affected teeth have a normal clinical appearance and are vital. Jaw hypoplasia and delayed eruption often result in a unilateral posterior open bite [16].

In addition, temporomandibular joint dysfunction, as evidenced by mandibular flexion towards the affected side and presenting malocclusion such as unilateral posterior open bite, highlights the need for comprehensive evaluation and management of temporomandibular joint disorders in patients with Parry-Romberg syndrome.

Treatment of atrophy is generally based on remodeling the adipose tissue destroyed by the condition [17]. Several therapeutic options are available, including autogenous fat grafts, cartilage grafts, silicone injections, the use of bovine collagen, and inorganic implants [18]. These therapeutic options are mainly intended to improve the aesthetic appearance of the face and the psychological comfort of patients. A multidisciplinary approach is often required, involving plastic surgeons, pediatric dentists, speech therapists, and psychologists [19,20]. Orthodontic treatment and prosthetic rehabilitation can help to resolve the aesthetic, functional, and psychological problems associated with this condition.

## Conclusions

The present case highlights the importance of recognizing the various symptoms associated with Parry-Romberg syndrome, particularly the oral manifestations, and the need for comprehensive, multidisciplinary management of the aesthetic and functional aspects of this condition. Further research is needed to better understand the pathophysiology of the oral manifestations of Parry-Romberg syndrome and optimize treatment strategies for affected individuals, to improve their quality of life.
